# Optimising Exercise for Managing Chemotherapy-Induced Peripheral Neuropathy in People Diagnosed with Cancer

**DOI:** 10.3390/cancers17152533

**Published:** 2025-07-31

**Authors:** Dhiaan Sidhu, Jodie Cochrane Wilkie, Jena Buchan, Kellie Toohey

**Affiliations:** 1 Physical Activity, Sport and Exercise Research Theme, Faculty of Health, Southern Cross University, Gold Coast 4225, Australia; dhiaan.sidhu@scu.edu.au (D.S.); jodie.cochrane.wilkie@scu.edu.au (J.C.W.); jena.buchan@scu.edu.au (J.B.); 2 Exercise Medicine Research Institute, School of Medical and Health Science, Edith Cowan University, Joondalup 6027, Australia; 3 Faculty of Health, University of Canberra, Canberra 2617, Australia

**Keywords:** cancer, CIPN, chemotherapy, exercise, physical activity

## Abstract

Background: Chemotherapy-induced peripheral neuropathy is a common and debilitating side effect of cancer treatment. While exercise has shown promise in alleviating this burden, it remains underutilised in clinical practice due to the lack of accessible, clinician-friendly guidance. Aim: This review aimed to synthesise current evidence on exercise interventions for managing chemotherapy-induced peripheral neuropathy and provide practical insights to support clinicians in integrating these approaches into patient care. Methods: A search was conducted across MEDLINE, CINAHL, and SPORTDiscus using keywords related to exercise and CIPN. Studies were included if they involved adults receiving neurotoxic chemotherapy and exercise-based interventions. Two authors independently screened studies and resolved conflicts with a third author. Study quality was assessed using the JBI Critical Appraisal Tools, and only studies meeting a minimum quality standard were included. A balanced sampling approach was employed. Data on study design, participant characteristics, interventions, and outcomes were extracted. Results: Eleven studies were included, covering various exercise modalities: multimodal (n = 5), yoga (n = 2), aerobic (n = 1), resistance (n = 1), balance (n = 1), and sensorimotor (n = 1). Exercise interventions, particularly multimodal exercise, significantly improved symptom severity, functionality, and quality of life (*p* < 0.05). The studies had high methodological quality, with randomised controlled trials scoring between 9/13 and 11/13, and quasi-experimental studies scoring 8/9 on JBI tools. Conclusions: This review highlights the significant benefits of exercise, especially multimodal exercise, for managing CIPN and provides guidance for integrating these strategies into clinical practice. Future research is needed to refine exercise prescriptions and develop standardised guidelines.

## Simple Summary

Chemotherapy-induced peripheral neuropathy (CIPN) is a painful and disabling condition experienced by many individuals undergoing cancer treatment. Despite growing evidence that exercise can help reduce its symptoms and improve quality of life, clear and practical guidance for healthcare providers is lacking. This study explored various exercise methods to better understand their effects on CIPN and to identify effective strategies for integrating exercise into patient care. By synthesising current research and offering actionable recommendations, the authors aim to bridge the gap between scientific evidence and clinical practice, encouraging the use of exercise as a tool to alleviate the burden of CIPN. The findings could inspire further research and aid clinicians in improving care for individuals coping with this challenging side effect of chemotherapy.

## 1. Introduction

Chemotherapy-induced peripheral neuropathy (CIPN) is a common neurological side effect caused by exposure to certain chemotherapy drugs, particularly taxanes, vinca alkaloids, platinum agents, proteasome inhibitors, and thalidomide [1]. These agents cause neurotoxicity through mechanisms such as neuroinflammation, microtubule disruption, altered ion channel activity, and damage to the DNA, mitochondria, and myelin sheath [2]. Approximately 68% of individuals receiving neurotoxic chemotherapy develop CIPN within the first month of treatment [3,4].

CIPN manifests as sensory symptoms such as pain, tingling, or numbness in the hands and feet or motor symptoms such as reduced deep tendon reflexes, ataxia, muscle weakness, and impaired balance [5]. While some individuals may experience mild or transient symptoms, others may suffer from severe or long-lasting symptoms that significantly impact their daily functioning [6] and quality of life [7].

CIPN is associated with additional challenges beyond physical symptoms, such as poor sleep quality and fatigue [8], psychological distress [9], a reduced sense of independence [9], impaired fine and gross motor skills [9], and an increased risk of injury and falls [10]. This can reduce chemotherapy tolerability [11], often resulting in treatment interruptions, dose reductions, or premature discontinuation, potentially jeopardising treatment effectiveness and survival outcomes [12].

Exercise is now firmly established within standard cancer care [13], with substantial evidence supporting its safety and effectiveness in managing cancer-related complications [14]. Exercise and Sports Science Australia (ESSA) has developed evidence-based exercise guidelines tailored to this population’s unique needs [15]. Translating research into clinical practice has resulted in tangible improvements in the lives of people living with cancer. However, these guidelines, as with all existing exercise guidelines, do not specifically address CIPN.

Exercise holds potential to alleviate both the physiological and psychological burdens of CIPN. Research indicates that this benefit may arise through mechanisms such as increased expression of neurotrophic factors, reduction of oxidative markers, increased release of anti-inflammatory cytokines, improved mood, reduced distress, and increased self-efficacy [16].

Despite these benefits, exercise remains underutilised in the clinical management of CIPN. This is due to the absence of tailored exercise guidelines [16] and the lack of accessible, clinician-friendly resources that translate existing evidence into actionable strategies. Many individuals with CIPN avoid exercise altogether due to limited guidance, fear of exacerbating symptoms, or insufficient support from healthcare providers. The lack of established CIPN-specific exercise guidelines, limited guidance for clinicians, uncertainty regarding exercise approaches, and the lack of referrals to qualified exercise professionals contribute to continued poor health outcomes and missed opportunities to improve care for this population.

Several reviews have explored the role of exercise in managing CIPN, but most have focused exclusively on randomised controlled trials (RCTs) [17,18]. While RCTs are the gold standard for establishing causality, limiting reviews to RCTs can result in overlooking useful findings from other study designs that investigate a wider range of exercise modalities, such as yoga and sensorimotor exercise. Despite contributing valuable evidence, previous reviews generally did not provide practical, clinician-focused resources to facilitate implementation in clinical settings.

This narrative review aimed to address these gaps by providing a comprehensive overview of exercise interventions for managing CIPN, examining various exercise modalities, frequencies, intensities, and durations, and their impact on patient outcomes. Including exercise prescriptions and outcome measures that have been previously overlooked, it offers an up-to-date snapshot of the CIPN and exercise research landscape. It also identifies promising approaches, highlights current best practices, guides future research, and provides practical insights and resources to support clinicians in translating research findings into real-world applications.

## 2. Methods

A literature search was conducted across MEDLINE, CINAHL, and SPORTDiscus electronic databases from inception to July 2024 using the search string: (exercis* OR “exercise prescription” OR “physical activit*” OR aerobic* OR “resistance training” OR “strength training” OR “movement training” OR “functional training” OR “balance training” OR yoga OR “sensorimotor training” OR Pilates OR rehabilitat* OR “physical therap*”) AND (“chemotherapy-induced peripheral neuropathy” OR CIPN OR “cancer related peripheral neuropathy” OR “chemotherapy-induced peripheral neurotoxicity” OR “chemotherapy-induced polyneuropathy”). All retrieved studies were independently screened by two authors against the inclusion criteria: (1) participants aged 18 years and older; (2) participants who had received or were undergoing neurotoxic chemotherapy; and (3) interventions with an exercise intervention. Studies were excluded if they met any of the following criteria: (1) participants under 18 years of age; (2) participants with peripheral neuropathy of other aetiologies (e.g., diabetic neuropathy); or (3) interventions that did not exclusively involve exercise. Conflicts in screening were resolved by a third author. Subsequently, studies were assessed for methodological quality using the Joanna Briggs Institute (JBI) Critical Appraisal Tools [19,20,21], completed independently by two authors, with disagreements resolved by a third author. Only studies meeting a minimum quality standard were considered for inclusion. Among these, a balanced sampling approach was applied to ensure representation across different exercise modalities, cancer types, and study designs. Specifically, 2–3 studies were selected for each exercise modality to provide a comprehensive overview. Data were extracted from included studies, including study design, participant characteristics, exercise interventions, and outcomes, to ensure academic and clinical rigor when providing recommendations. This process ensured that the narrative review captures the breadth of exercise interventions studied within this population.

## 3. Results

**Figure 1:**
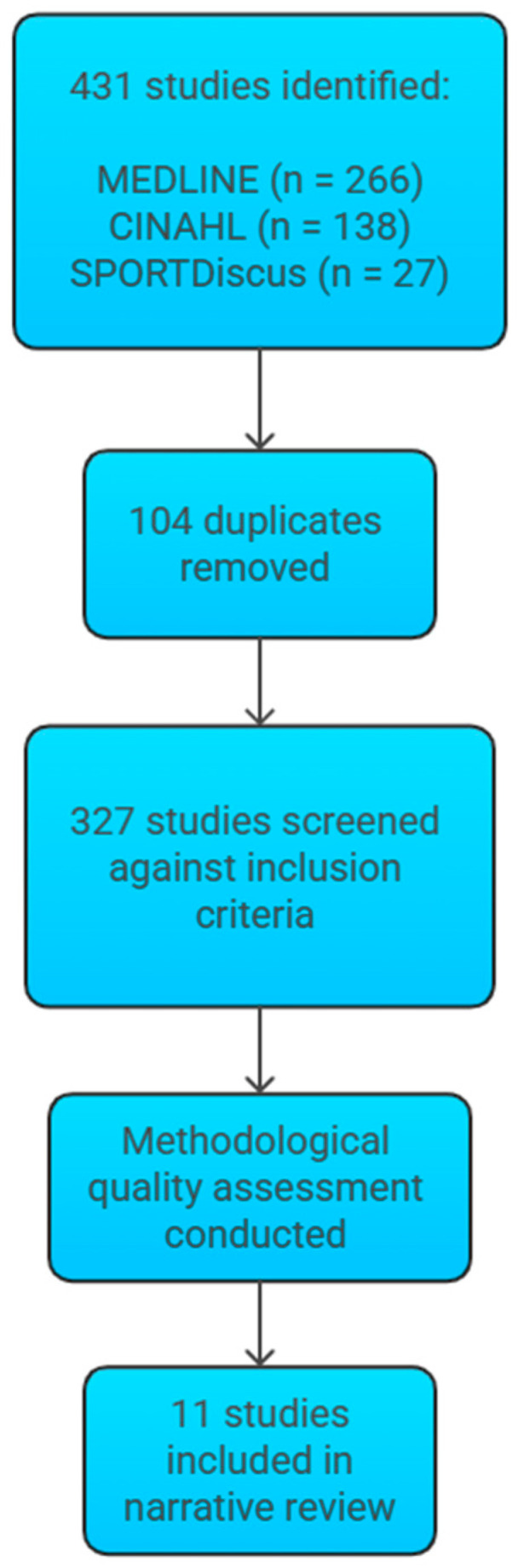
A flow diagram created using Napkin AI (version beta.0.13.2) illustrating the study selection process for the narrative review. A total of 431 studies were identified (MEDLINE = 266, CINAHL = 138, SPORTDiscus = 27). After removing duplicates, 327 records remained and were screened against the inclusion criteria. Following methodological quality assessment, 11 studies were included in the narrative review.

Eleven studies were included in this narrative review, representing a total of 462 participants. Figure 1 outlines the study selection process for this narrative review.

Of these studies, four had only female participants, while the others included males and females. A range of cancer types were represented, including breast, uterine, ovarian, colon, bladder, colorectal, hematologic, gastrointestinal, gynaecological, non-Hodgkin’s and Hodgkin lymphoma, melanoma, prostate, pancreas, chronic lymphocytic leukaemia, adenocarcinoma, pancreatic, plasmacytoma, multiple myeloma, rectal, and lung cancers. The ages of participants ranged from approximately 20 to 89 years (Table S1 (see Supplementary Materials)). The exercise modalities comprised multimodal (n = 5), yoga (n = 2), aerobic (n = 1), resistance (n = 1), balance (n = 1), and sensorimotor (n = 1) (Table 1). This reflects the wide range of exercise modalities of the included studies. The JBI critical appraisal scores for the RCT studies (n = 9) ranged between 9/13 and 11/13 (Table 2). The quasi-experimental studies (n = 2) scored 8/9 (Table 3). One of these quasi-experimental studies also included a qualitative component and was additionally appraised using the qualitative study tool, scoring 7/10 (Table 4). These scores reflect the high methodological quality of the included studies.

The Joanna Briggs Institute (JBI) scores provide an indication of the methodological quality of the included studies, with higher scores reflecting stronger adherence to best-practice research standards. For example, scores ranging from 9/13 to 11/13 suggest that most key criteria for methodological rigor were met, but there may still be some limitations or areas where potential bias could be present. While these scores do not indicate a high risk of bias, they do highlight the importance of interpreting findings with some caution, particularly for studies that did not achieve the maximum score.

## 4. Discussion

This review highlighted the potential benefits of incorporating aerobic, resistance, balance, sensorimotor, and yoga exercises into standard care for CIPN. It showed that significant improvements were seen in symptom severity, quality of life, and functional capacity among patients engaging in these exercise modalities. The findings and recommendations from this review are presented below, divided into exercise modalities for ease of clinical application.

### 4.1. Multimodal Exercise in the Management of CIPN

This review highlights the potential benefits of various multimodal exercise interventions in managing chemotherapy-induced peripheral neuropathy (CIPN). Notably, five of the eleven included studies [22,23,24,25,26] demonstrated that a combination of aerobic, resistance, balance, and sensorimotor exercises can lead to significant improvements in symptom severity, quality of life, and functional capacity among people suffering from CIPN symptoms. It was found that engaging in these exercise modalities may mitigate the debilitating effects of CIPN, such as pain, weakness, and sensory disturbances, thus enhancing overall patient well-being. Despite these promising outcomes, the variability in participant demographics, exercise protocols, cancer treatments, and assessment tools across studies reduces the generalisability of the results. Interestingly, while most of the participants would be meeting the resistance exercise guidelines recommended for the general or oncology population [33], many of them would not meet the aerobic guidelines [13,15,33]. This is an important finding as it highlights that significant benefits may still be attained below recommended thresholds, emphasising that even small levels of exercise can result in meaningful improvements. Multimodal exercise appears to be the preferred modality in this population, consistent with trends observed in the general population. More targeted research is needed to determine the optimal exercise combinations and to establish guidelines for implementing such interventions in clinical practice.

### 4.2. Aerobic Exercise in the Management of CIPN

Aerobic exercise has been widely recognised for its health benefits, particularly in improving cardiovascular fitness, which is critical for people undergoing cancer treatment. The current evidence supporting aerobic exercise for managing CIPN is still emerging, with the study by Cao et al. [29] representing the only addition to this body of literature. The findings suggest that a structured aerobic regimen can lead to meaningful improvements in CIPN symptom severity, particularly in a sizeable female cohort. However, the reliance on self-reported outcomes raises the need for studies that incorporate objective functional assessments to strengthen the evidence base. This exercise prescription coincides with the guidelines recommended for the general and oncology population of at least 150 min of moderate-intensity physical activity throughout the week [13,15,33]. Despite the promising results, the lack of studies focusing exclusively on aerobic exercise in diverse cancer populations highlights a gap in understanding how different aerobic modalities may cater to various needs and capabilities of individuals with CIPN.

### 4.3. Resistance Exercise in the Management of CIPN

Resistance exercise is of growing interest due to its potential to increase muscle strength, which is often compromised in individuals with CIPN. Chen et al. [30] provided the only insight into low-intensity resistance training as a viable option for people who may be unable to engage in higher-intensity exercise due to their condition. The study found that resistance training improves function and quality of life in those receiving oxaliplatin [30]. The limited sample size and absence of randomisation reduce the generalisability of the findings. The exercise prescription aligns with the guidelines recommended for the general and oncology populations of performing muscle-strengthening exercises on at least 2 days per week [13,15,33]. Research on resistance exercise for CIPN remains limited, necessitating more robust studies to establish clear guidelines. Furthermore, exploring the safety and efficacy of higher-intensity resistance training for individuals with CIPN could provide a broader perspective on how to optimise strength training interventions.

### 4.4. Balance and Sensorimotor Training in the Management of CIPN

Balance and sensorimotor training addresses critical functional impairments that arise in individuals with CIPN [31,32]. These impairments can significantly increase the risk of falls and injuries, thus compromising the safety and independence of these individuals. Current evidence from studies by Schwenk et al. [31] and Streckmann et al. [32] suggests that balance training using innovative technology can improve postural stability and balance metrics. These studies show how technological advancements can expand exercise prescription. However, the lack of detailed exercise prescriptions and standardised measures across studies complicates comparisons and the development of guidelines. Due to the safety considerations and required equipment, both interventions took place in a supervised environment, which may limit their accessibility [31,32]. However, this does not preclude the feasibility or potential effectiveness of home-based or unsupervised alternatives. More research is warranted to develop specific recommendations for balance and sensorimotor training tailored to the unique needs of people with CIPN. Further investigations incorporating larger sample sizes and objective measures will improve the understanding of these interventions’ effectiveness.

### 4.5. Yoga in the Management of CIPN

Yoga reduces the physical and emotional burdens associated with CIPN by promoting relaxation, flexibility, and strength. Studies by Galantino et al. [28] and Bao et al. [27] provide initial evidence suggesting yoga’s benefits for individuals experiencing CIPN, such as improved quality of life and physical function, and reduced symptom severity. However, there is an inconsistency in exercise prescriptions and standard assessments for evaluating effectiveness. While RCTs are considered to be the gold standard of evidence to inform and support clinical decision-making and evidence-based healthcare [34], qualitative research allows for the voices and lived experiences of people with CIPN to be prioritised, aligning with the principles of patient-centred care. Although these studies imply that yoga may be a beneficial intervention, the small sample sizes and varied methodologies limit the robustness of the conclusions. Larger, more rigorous randomised controlled trials are required to establish yoga as a standard exercise modality in CIPN management and to identify optimal practices for clinical application.

### 4.6. Key Findings

Table 5 summarises the key findings from this review, highlighting the positive effects of various exercise modalities on CIPN, the lack of standardised exercise guidelines, key research gaps, and the need for larger, more robust studies to strengthen the evidence base. While this review presents exercise as a promising strategy for managing CIPN, it is important to acknowledge that not all studies reported consistently positive results. In some instances, improvements in outcomes such as symptom severity [23], quality of life [24,25], and gait speed [31] were not statistically significant.

### 4.7. Strengths and Limitations

A key strength of this narrative review is its inclusion of studies with varying study designs, providing a comprehensive overview of exercise modalities for managing CIPN. Studies overlooked in previous reviews were included, which revealed promising interventions not yet tested in RCTs. This offers guidance for future research. Most of the included studies had a high level of methodological quality, which increases the reliability and validity of the findings. The format of this review provides clinicians with a clear and accessible resource to guide the implementation of evidence-based care for CIPN.

The primary limitations of this narrative review stem from the lack of depth and the inability to support or compare studies effectively due to the limited number of studies, heterogeneity of study designs, populations, and outcome measures. The considerable heterogeneity also complicates synthesis, reduces generalisability, and increases uncertainty in interpreting the evidence. Several studies had potential bias due to the lack of blinding, control groups, allocation concealment, and possible researcher influence. It is important to acknowledge that narrative reviews are unable to draw definitive conclusions about the effectiveness of exercise modalities.

### 4.8. Practical Applications

**Figure 2:**
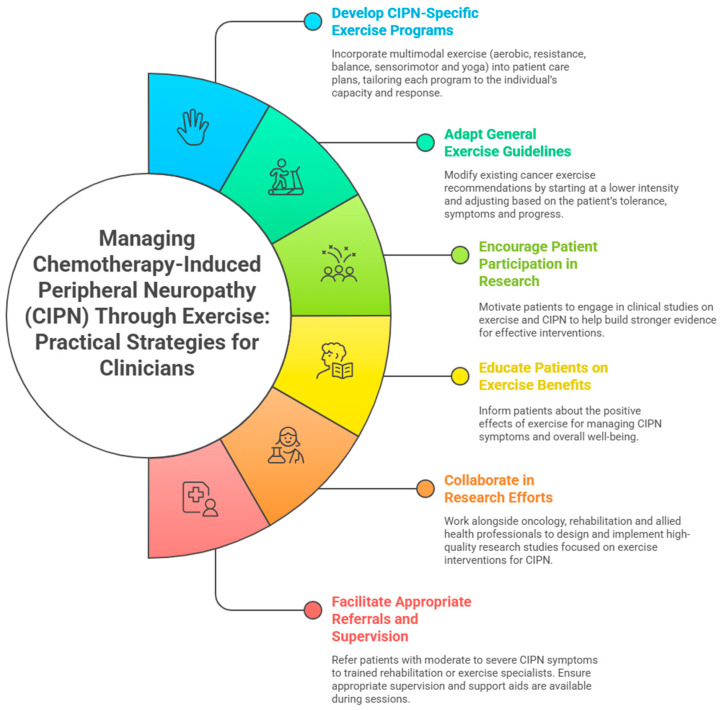
An infographic created using Napkin AI (version beta.0.13.2) outlining six practical steps clinicians can take to support individuals with chemotherapy-induced peripheral neuropathy (CIPN) through exercise: (1) developing tailored, multimodal exercise plans; (2) adapting general exercise guidelines; (3) educating patients on exercise benefits; (4) encouraging participation in research; (5) collaborating in research efforts; (6) facilitating appropriate referrals and supervision.

Table 6 and Figure 2 outline the practical applications based on the findings of this review, providing clinicians with actionable guidance for managing patients with CIPN and assisting in the translation of research findings into clinical practice.

### 4.9. Future Research Directions

This review highlights the need for additional research in exercise for CIPN management to determine the optimal exercise prescription for CIPN management, particularly randomised controlled trials with large sample sizes. Future research should address gaps such as the limited evidence on isolated resistance and aerobic exercise, and the variability in yoga protocols, which reduces comparability. Incorporating participant feedback and conducting meta-analyses will play a key role in developing evidence-based exercise guidelines for people with CIPN. To progress this work, future research should also consider holistic approaches across interdisciplinary teams to minimise the impact of CIPN and improve its management for people diagnosed with cancer.

## 5. Conclusions

This narrative review found that exercise may provide significant benefits for people with CIPN. The evidence presented emphasises the potential of multimodal exercise interventions as a vital component in the management of CIPN. Given the benefits demonstrated across various exercise modalities, integrating structured aerobic, resistance, balance, sensorimotor, and yoga exercises into patient care plans could significantly improve CIPN symptoms, quality of life, and functional outcomes. These findings align with improved oncology outcomes, supporting the integration of exercise into routine cancer care. Clinicians should consider incorporating these exercise programs into routine care to enhance patient recovery and well-being, with further research needed to refine prescriptions and optimise intervention effectiveness.

## Figures and Tables

**Table 1:** Clinician-focused summary of exercise prescription and benefits for CIPN.

Exercise Modality	Study	Exercise Prescription	Benefits
Multimodal	Bland et al., 2019 [22]	Type: Aerobic—cycle ergometer/walking Resistance—full body exercises using machines, free weights, and resistance bands Balance—progressively more difficult exercises on progressively unstable surfaces Frequency: five days per week Intensity: Aerobic—50–75% heart rate reserve/12 to 14 on Borg Scale Resistance—50–65% of 1 repetition maximum Time: Aerobic—40 min	↓ Symptom severity ↑ Quality of life ↑ Chemotherapy completion rate
	Ikio et al., 2022 [23]	Type: Resistance—grip and pinching movements Manual dexterity—origami and paper tearing Sensory function—material identification and tactile perception practice Frequency: at least 3 times per week Intensity: 40–60% of maximum muscle strength Time: 30 min	↑ Strength ↑ Upper extremity function
	Kneis et al., 2019 [24]	Type: Aerobic—stationary bike Balance—progressively reducing support surface and visual input, adding motor/cognitive tasks, and instability induction Frequency: twice per week Intensity: moderate intensity/below the individual anaerobic threshold Time: 60 min	↑ Balance ↓ Symptom severity ↑ Cardiorespiratory fitness
	Vollmers et al., 2018 [25]	Type: Sensorimotor—static and dynamic single-leg stance and smovey exercises on posturomed devices Resistance—full body exercises Aerobic—warm-up Frequency: twice per week Intensity: RPE of 13–15 on the Borg Scale	↑ Strength ↑ Balance
	Zimmer et al., 2018 [26]	Type: Balance—balance pads, balancing on lines Coordination—cherry pit pillows, Brasils Aerobic—cross trainer/bicycle ergometer/walking Resistance—circuit-based full body exercises Stretching—cool-down Frequency: twice per week Intensity: Aerobic—RPE of 12–13 on Borg scale/60–70% of maximum heart rate Resistance—Borg CR10 scale level 6/60–80% of 1 repetition maximum Time: 60 min	↑ Balance ↑ Strength ↓ Symptom severity ↑ Quality of life
Yoga	Bao et al., 2020 [27]	Type: breathwork and modifiable postures Frequency: daily Time: 60 min	↓ Pain ↑ Quality of life ↑ Stability/balance ↑ Physical performance
	Galantino et al., 2019 [28]	Type: somatic seated and supine movements Frequency: twice per week Time: 180 min per week	↑ Flexibility ↑ Balance ↑ Physical function ↓ Pain ↑ Vibration sense ↓ Symptom severity
Aerobic	Cao et al., 2023 [29]	Type: brisk walking Intensity: moderate Time: 150 min per week	↓ Symptom severity
Resistance	Chen et al., 2020 [30]	Type: lower-limb elastic band exercises Frequency: 3 per week Intensity: low intensity Time: 40 min	↑ Strength ↑ Aerobic endurance ↓ Symptom severity ↑ Quality of life
Balance	Schwenk et al., 2016 [31]	Type: interactive, sensor-based, cognitively challenging, dynamic weight-shifting tasks Frequency: twice per week Time: 45 mins	↑ Balance
Sensorimotor	Streckmann et al., 2019 [32]	Type: progressively more difficult balance exercises on progressively unstable surfaces Frequency: twice per week Time: each exercise performed 3 times for 20 s with a rest of 40 s between each set and 1 min between each exercise	↓ Symptom severity

This table outlines various exercise modalities, corresponding studies, and their detailed exercise prescription (frequency, intensity, time, and type), along with their reported benefits. Key: ↑ = statistically significant increase, ↓ = statistically significant decrease.

**Table 2:** JBI critical appraisal of the included RCT studies.

Study	Criteria 1	Criteria 2	Criteria 3	Criteria 4	Criteria 5	Criteria 6	Criteria 7	Criteria 8	Criteria 9	Criteria 10	Criteria 11	Criteria 12	Criteria 13	Summary
Bao et al., 2020 [27]	Yes	Yes	Yes	No	No	Yes	No	Yes	Yes	Yes	Yes	Yes	Yes	10/13—potential bias due to lack of blinding of participants, assessors, and individual delivering treatment
Bland et al., 2019 [22]	Yes	Yes	Yes	No	No	Yes	No	Yes	Yes	Yes	Yes	Yes	Yes	10/13—potential bias due to lack of blinding of participants, assessors, and individual delivering treatment
Cao et al., 2023 [29]	Yes	Unclear	Yes	No	No	Yes	Unclear	Yes	Yes	Yes	Yes	Yes	Yes	9/13—potential bias due to lack of blinding of participants and individual delivering treatment, and insufficient information on assessor blinding and allocation concealment
Ikio et al., 2022 [23]	Yes	Yes	Yes	No	N/A	Yes	Yes	Yes	Yes	Yes	Yes	Yes	Yes	11/13—minimal bias due to lack of participant blinding
Kneis et al., 2019 [24]	Yes	Yes	Yes	No	No	Yes	Unclear	Yes	Yes	Yes	Yes	Yes	Yes	10/13—potential bias due to lack of blinding of participants and individual delivering treatment, and insufficient information on assessor blinding
Schwenk et al., 2016 [31]	Yes	Yes	Yes	No	No	Yes	Yes	Yes	Yes	Yes	Yes	Yes	Yes	11/13—minimal bias due to lack of blinding of the participant and individual delivering treatment
Streckmann et al., 2019 [32]	Yes	Yes	Yes	No	No	Yes	Yes	Yes	Yes	Yes	Yes	Yes	Yes	11/13—minimal bias due to lack of blinding of the participant and individual delivering treatment
Vollmers et al., 2018 [25]	Yes	Unclear	Yes	No	No	Yes	Unclear	Yes	Yes	Yes	Yes	Yes	Yes	9/13—potential bias due to lack of blinding of participants and individual delivering treatment, and insufficient information on assessor blinding and allocation concealment
Zimmer et al., 2018 [26]	Yes	Yes	Yes	No	No	Yes	No	Yes	Yes	Yes	Yes	Yes	Yes	10/13—potential bias due to lack of blinding of participants, assessors, and individual delivering treatment

Each criterion is rated as “Yes”, “No”, “Unclear”, or “N/A”. The summary column includes the total number of criteria met and a brief description of potential bias.

**Table 3:** JBI critical appraisal of the included quasi-experimental studies.

Study	Criteria 1	Criteria 2	Criteria 3	Criteria 4	Criteria 5	Criteria 6	Criteria 7	Criteria 8	Criteria 9	Summary
Chen et al., 2020 [30]	Yes	No	Yes	Yes	Yes	Yes	Yes	Yes	Yes	8/9—minimal bias due to the lack of a control group
Galantino et al., 2019 [28]	Yes	No	Yes	Yes	Yes	Yes	Yes	Yes	Yes	8/9—minimal bias due to the lack of a control group

Each criterion is rated as “Yes” or “No”. The summary column includes the total number of criteria met and a brief description of potential bias.

**Table 4:** JBI critical appraisal of the included qualitative studies.

Study	Criteria 1	Criteria 2	Criteria 3	Criteria 4	Criteria 5	Criteria 6	Criteria 7	Criteria 8	Criteria 9	Criteria 10	Summary
Galantino et al., 2019 [28]	Unclear	Yes	Yes	Yes	Yes	No	No	Yes	Yes	Yes	7/10—potential bias due to insufficient information on philosophical perspectives and potential influence of the researchers’ beliefs and values

Each criterion is rated as “Yes”, “No” or “Unclear”. The summary column includes the total number of criteria met and a brief description of potential bias.

**Table 5:** Key findings of this review.

Key Findings	Details
Positive impact of exercise on CIPN	Exercise, including aerobic, resistance, balance, sensorimotor, and yoga, showed improvements. Multimodal exercise is recommended. Benefits include improvements in symptom severity, quality of life, chemotherapy completion rate, strength, function, balance, and aerobic fitness.
Lack of standardised guidelines	Absence of established CIPN-specific exercise guidelines. Studies are varied in design, population, and exercise prescriptions, making it challenging to identify a definitive, evidence-based approach.
Research gaps	Optimal exercise prescriptions for CIPN management and the physiological mechanisms underlying exercise-induced changes in CIPN.
Need for robust studies	The field is relatively under-researched, and there is a need for more RCTs with larger sample sizes.

This table summarises the positive effects of exercise, research gaps, and recommendations for future studies.

**Table 6:** Practical applications of this review (illustrated in Figure 2).

Practical Applications	Details
Developing CIPN-specific guidelines	Incorporate multimodal exercise regimens (aerobic, resistance, balance, sensorimotor, and yoga exercises) for patients with CIPN. Tailor exercise based on individual capacity and response.
General exercise guidelines for CIPN patients	Adapt general cancer exercise recommendations cautiously. Start at a lower intensity and progress based on tolerance and patient response.
Encouraging patient participation in future research	Encourage patients to participate in research studies to increase sample sizes and obtain more diverse data for better evidence and precise exercise guidelines.
Integrating balance and sensorimotor training	Emphasise balance and sensorimotor exercises in rehabilitation programs to enhance stability, reduce fall risk, and improve neuromuscular function.
Collaborative research efforts	Healthcare institutions, oncology clinics, and rehabilitation centres could collaborate for high-quality randomised controlled trials on CIPN and exercise.
Facilitate referrals and supervision	Clinicians should refer patients with CIPN, especially those with severe symptoms or high fall risk, to rehabilitation or exercise specialists for tailored programs. Supervision and use of support aids are recommended to reduce fall risk and enhance safety.

This table outlines strategies for implementing exercise programs, encouraging patient involvement, and promoting collaborative research efforts.

## Supplementary Materials

The following supporting information can be downloaded at: https://www.mdpi.com/article/10.3390/cancers17152533/s1, Table S1: Comprehensive summary of the included studies [22,23,24,25,26,27,28,29,30,31,32].
