# Supplementary material for: Optimising Exercise for Managing Chemotherapy-Induced Peripheral Neuropathy in People Diagnosed with Cancer
Source: Cancers (Basel). 2025 Jul 31;17(15):2533. doi: 10.3390/cancers17152533 (PMC12346131; doi:10.3390/cancers17152533)
Supplement: Supplementary file 1 [file cancers-17-02533-s001.zip › cancers-3738231-supplementary.pdf]

Supplementary Materials

Table S1: Comprehensive summary of the included studies. This table outlines the author and year, study characteristics, participant characteristics, intervention characteristics, outcome measures and results.

Author and Year	Study Characteristics	Participant Characteristics	Intervention Characteristics	Outcome Measures	Results
[27] Bao et al., 2020	Pilot randomised wait-list controlled trial<br>Sample size: n = 41	Females<br>Age: 35.5-79 years<br>Type of cancer: breast, uterine, ovarian	Type: yoga- breathwork and modifiable postures<br>Frequency: daily<br>Intensity: N/A<br>Time: 60mins, 8-weeks<br>Supervised in-person group class twice per week<br>Unsupervised at-home five times per week	Symptom severity- Numeric Rating Scale (NRS)<br>Quality of life- Neurotoxicity subscale of the Functional Assessment of Cancer Therapy/Gynecologic Oncology Group Neurotoxicity Questionnaire (FACT/GOG-Ntx)<br>Stability/balance- The Functional Reach Test (FR)<br>Physical performance- Chair to stand (CTS)<br>Speed- 4m walk test (4MWT)	NRS- At week 8 from baseline, mean NRS pain decreased by 1.95 points (95% CI = -3.20 to -0.70) in the yoga arm, and by 0.65 points (-1.81, 0.51) in the usual care arm; *P* = .14. At week 12 from baseline, mean NRS pain decreased by 2.03 points (95% CI = -3.25 to -0.80) in the yoga arm, and by 0.08 points (95% CI = -1.27 to 1.10) in the usual care arm; *P* = .026. No statistically significant change in other NRS scores.<br>FACT/GOG-Ntx- At week 8, it increased by 4.25 (95% CI = 2.29 to 6.20) in the yoga group vs 1.36 (95% CI = -0.47 to 3.19) in the usual care group; *P* = .035.<br>FR- mean functional reach was similar in the 2 treatment groups at baseline; by 8 weeks it has increased by 7.14 cm (95% CI = 3.68 to 10.59) in the yoga group and decreased by 1.65 cm (95% CI = -5.00 to 1.72) in the usual care

					group (P = .001 for the difference between groups).<br><br>CTS- time improved by 3.65 seconds (95% CI = -4.86 to -2.4) in the yoga group compared with 1.55 seconds (95% CI = -2.67 to -0.43) in the usual care group (P = .013 for the difference between groups)<br><br>4MWT- no statistically significant differences
[22] Bland et al., 2019	2-arm randomised controlled trial<br><br>Sample size: n = 27	Females<br><br>Mean age (SD): 50.2 (10.2)<br><br>Type of cancer: breast	Type: Multimodal program:<br>Aerobic exercise: treadmill, cycle ergometer or walking outside<br>Resistance exercise: 5 exercises using machines, free weights, or resistance bands to target the primary upper and lower body muscle groups starting at 1 set of 10 repetitions and progressing to 2 sets of 10-12 repetitions. Hand and foot exercises- resisted hand opening and closing, resisted wrist flexion and extension and resisted foot and ankle movements. Core exercises- glute bridge and core activation with knee fall-out progressing to bird-dog and dead bug with Swisse ball.<br>Balance training: 2 single leg standing exercises for 6 to 8 repetitions (20-30 seconds per exercise), with 1min of rest. Progressed from being performed on a stable surface with support to being performed unsupported on an unstable surface.<br><br>Frequency: five days per week (3 days of supervised resistance, aerobic and	Symptom severity- The European Organization for Research and Treatment of Cancer Quality of Life Questionnaire (EORTC QLQ) CIPN20 subscale<br><br>Non-automated Quantitative Sensory Testing- vibration sensation and temporal summation of pain (pinprick) performed at the lower limb<br><br>Quality of life- The European Organization for Research and Treatment of Cancer Quality of Life Questionnaire (EORTC QLQ-C30) core questionnaire.<br><br>Chemotherapy completion rate- number of participants who experienced a dose reduction or a complete treatment cancellation, relative dose intensity, planned time to complete chemotherapy and chemotherapy dose received and timing of administration.	Symptom severity: There were no differences between groups at any time point for CIPN20 symptom scores. There was a main effect of time for sensory and motor symptoms in both groups combined (P < .01). Mean sensory symptoms significantly increased from baseline to pre-cycle 4 (Δ16.8 ± 3.2, P < .01) and at the end of chemotherapy (Δ24.3 ± 4.6, P < .01). There was a further increase in symptoms post-chemotherapy compared to pre-cycle 4 (Δ7.3 ± 3.3, P = .04). At the follow-up, sensory symptoms recovered in both groups and were not different from baseline (Δ3.0 ± 1.9, P = .14). Relative to baseline, mean motor symptoms significantly increased in both groups at pre-cycle 4 (Δ6.5 ± 1.6, P < .01) and at the end of chemotherapy (Δ10.5 ± 1.9, P < .01). There was a significant change from pre-cycle 4 to the end of chemotherapy (Δ3.9 ± 1.6, P = .02). Motor symptoms remained higher for both groups in the follow-up than at baseline (Δ5.3 ±

			balance exercise, and 2 days of home-based aerobic exercise)<br><br>Intensity:<br>Aerobic exercise:<br>Supervised- For the week after chemotherapy- 50%-55% heart rate reserve. After the first week of the chemotherapy cycle- increased to 75% heart rate reserve by week 8.<br>Home-based- 12 to 14 on Borg Scale.<br>Resistance exercise: starting at 50% of estimated 1 repetition maximum, progressing to 65% of 1 repetition maximum.<br><br>Time: 8–12-week program<br>Aerobic exercise:<br>Supervised- starting with 40mins during chemotherapy. Progressed from 25-35mins on non-chemotherapy weeks.<br>Home-based- progressing from 15-30mins.<br><br>Supervised 3 days a week, home-based 2 days a week<br><br>Note: Immediate exercise (IE) group- exercise intervention occurred during chemotherapy, followed by a period of no formal exercise training<br>Delayed exercise (DE) group- usual care during chemotherapy, followed by identical exercise intervention after chemotherapy.		1.9, $P < .01$). There were no significant changes over time for autonomic symptoms. The percentage of participants reporting moderate to severe numbness in toes or feet was lower in the IE group compared to the DE group at pre-cycle 4 (IE: n = 1, 9%, DE: n = 7, 50%, $P = .04$). However, they were similar by the end of chemotherapy (IE: n = 5, 42%; DE: n = 8, 57%, $P = 1.0$). There were no other differences between groups in patient-reported moderate to severe symptoms for the CIPN20 sensory items.<br><br>Non-automated quantitative sensory testing: At baseline, 4 participants (DE: n = 3, IE: n = 1) exhibited impaired vibration sense. At pre-cycle 4, there was a significant difference between groups in the percentage of participants exhibiting impaired vibration sense (IE: n = 2, 18%, DE: n = 10, 83%, $P < .01$). There was a similar trend in toe/foot numbness. However, by the end of chemotherapy and at follow-up, there were no significant differences between groups (end of chemotherapy: $P = .71$, follow-up: $P = .13$). No differences between groups were found for vibration sense at medial malleolus and patella or for the pinprick test at any time point.<br><br>Quality of life: There was an interaction between group and time for the EORTC QLQ-C30 overall global health status/quality of life ($P = .01$). Overall quality of life was greater in the IE group compared to the DE by

					the end of chemotherapy (mean difference = 11.9, *P* = .05). Upon completion of the exercise intervention, overall quality of life increased by a mean of 18.5 points in the DE group, while it was unchanged in the IE group. No significant difference between groups was observed at follow-up (*P* = .29). There were no other significant group by time interactions for the other subscales. There were significant main effects of time for physical function, cognitive function, and emotional function ($P < 0.05$). Significant changes across time points in both groups combined were found for overall quality of life and cognitive function ($P < 0.01$).<br><br>Chemotherapy completion rate- 10 participants experienced a dose reduction of at least 10% or a complete dose cancellation for one or more of their treatments (IE: n = 2, 20%; DE: n = 8, 53%, *P* = .06). 3 participants did not complete their treatment within the planned time frame (IE: n = 1, 8%, DE: n = 2, 13%, *P* = 1.0). Mean relative dose intensity did not significantly differ between groups (IE: 95.9 ± 6.1%, DE: 92.2 ± 11.0%, *P* = .31). However, all IE participants received at least the clinically relevant threshold of 85% relative dose intensity. Fewer DE participants reached this threshold (IE: n = 12, 100%, DE: n = 10, 67%, $P <$ .05).
[29] Cao et al.,2023	Secondary analysis of a multi-centre, open-	Females	Type: aerobic exercise- brisk walking	Symptom severity/pain: Functional Assessment of Cancer	The CIPN score was reduced from baseline by 1.3 (95% CI, −2.3 to −0.2)

	label, population-based, phase 3 randomized clinical trial<br>Sample size: n = 134	Mean age (SD): 57.5 (8.3)<br>Type of cancer: ovarian	Frequency: N/A<br>Intensity: moderate<br>Time: 150mins per week, 6-months<br>Home-based, facilitated by weekly phone calls	Therapy/Gynecologic Oncology Group–Neurotoxicity (FACT/GOG-Ntx)	points in the exercise intervention arm post-intervention.<br>No significant 6-month change in CIPN score was observed in the attention control arm (0.4; 95% CI, −0.8 to 1.5 points).<br>The between-group difference in CIPN score was −1.6 points (95% CI, −3.1 to −0.2 points; *P* = .03).
[30] Chen et al., 2020	Non-randomised preliminary study of one-group, pretest-post-test repeated measures, longitudinal design<br>Sample size: n = 42	Males and females: M = 20<br>F = 22<br>Age: 35-80 years<br>Type of cancer: colorectal	Type: resistance exercise- lower-limb elastic band<br>1) warm-up exercises lasting 4–5 min (consisting of 50 stationary stepping repetitions), forward and backward step exercises for both legs (20 repetitions) and left and right side step exercises (20 repetitions)<br>2) hip joint exercises lasting 6–8 min (consisting of back thigh resistance band exercises focusing on forward, backward, and sideward movements (8–10 8-s repetitions/direction)<br>3) knee joint exercises lasting 4–6 min (8–10 8 s repetitions forward and backward lower leg movements) 4) ankle joint exercises lasting 4–6 min (8–10 8 s repetition of upward and downward foot movements)<br>5) cool-down exercises lasting 4–5 min (identical to the warm-up exercises)<br>Frequency: 3 per week<br>Intensity: low-intensity, light resistance bands (yellow)<br>Time: 4.5-month program, 40mins<br>Home-based	Symptom severity- Common Terminology Criteria for Adverse Events version (CTCAE) v 4.03<br>Sensory function- using 128-Hz semi-quantitative tuning fork labelling<br>Motor function- manual muscle testing<br>Balance- tandem stance test and single leg stance test<br>Muscular strength- One-repetition maximum (IRM) leg extension<br>Aerobic endurance- 6-minute walking distance (6MWD)<br>Quality of life- The European Organization for Research and Treatment of Cancer Quality of Life Questionnaire-Chemotherapy-Induced Peripheral Neuropathy 20 (EORTC QLQ-CIPN20)<br>The 30-item disease-specific EORTC QLQ-Questionnaire-Core 30 (EORTC QLQ-C30)	CIPN prevalence increased gradually from 26.2% at T1, to 71.5% at T2 and 76.3% at T3. The majority of the participants experienced grade I symptom severity and none of them experienced grade III severity.<br>There were no evident negative changes in vibration sensational and muscle power as all participants were within normal ranges at all time points.<br>Abnormalities were seen in 2.4%, 9.5% and 7.7% of sample in tandem stance test from T1 to T3, respectively. Single leg stance abnormalities were observed in 16.7%, 19.1% and 15.4% of sample from T1 to T3, respectively.<br>Both the 1RM and 6MWD improved significantly at T2 and T3 relative to T1 (*P* < .001).<br>The participants achieved 1RM weight of 20.38 ± 5.91 kg in T1, 20.66 ± 6.01 kg in T2, and 20.97 ± 5.93 kg in T3.<br>The participants achieved an average of 435.57 ± 90.63 m in T1,

					463.26 ± 95.22 in T2, and 490.79 ± 87.36 m in T3 in 6MWD.<br><br>In the EORTC QLQ-CIPN20:<br>Sensory neurological symptoms increased gradually from 5.48 ± 6.06 (T1) to 7.64 ± 5.84 (T2) and 9.09 ± 5.98 (T3). There were significant increases in sensory symptoms as the number of chemotherapy cycles (T2, $P = .006$; T3, $P < .001$) increased relative to baseline (T1)<br>Motor neurological symptoms increased slightly from 1.19 ± 3.79 (T1) to 2.24 ± 7.46 (T2) and 2.28 ± 5.21 (T3). There was a statistically significant increase from T1 to T3 ($P = .049$), but not from T1 to T2 ($P = .130$).<br>Autonomic symptoms decreased gradually over time from 4.89 ± 10.13 (T1) to 2.91 ± 7.17 (T2) and 2.28 ± 5.21 (T3). They declined significantly from T1 to T3 ($P = .002$), but not from T1 to T2 ($P = .092$).<br><br>In the QOL-C30:<br>There were no significant changes in overall quality of life function between T1 to T2 ($P = .364$) or between T1 to T3 ($P = .556$).<br>There were gradual decreases in the impact on overall symptoms from 8.10 ± 9.90 at T1, 7.88 ± 8.94 at T2 and 5.65 ± 6.23 at T3. These declines were significant from T1 to T3 ($P = .039$), but not from T1 to T2 ($P = .860$).

					There were gradual improvements in global health, from 77.98 ± 11.61 at T2 to 75.85 ± 12.65 at T3. There were significant differences from T1 to T2 (*P* = .048), but not for T1 and T3 (*P* = .338).
[28] Galantino et al., 2019	Open-label, single-arm, mixed-methods feasibility trial<br>Sample size: n = 10	Males and females:<br>M = 1<br>F = 9<br>Age: 47-81 years<br>Type of cancer: ovarian, breast, colon, uterine, bladder	Type: somatic yoga-<br>1) Seated:<br>- Verbal check-in (5 minutes)<br>2) Supine:<br>- Body and breath scan, diaphragmatic breathing, intention setting (6 minutes)<br>3) Somatic Movements (15 minutes):<br>- Arch/flatten back, arch/curl, twists, hip series<br>- Knees to chest, knee circles (1 minute)<br>- Seated Sukasana (1 minute)<br>- Finger fan, chin mudra with kaki breath, sun breaths, and neck stretches (5 minutes)<br>- Dandasana (staff pose) joint freeing (4 minutes)<br>- Switch Sukasana (4 minutes)<br>- Side bends (2 minutes)<br>4) Marjariasana (table top) & Chakravakasana (4 minutes)<br>5) Tadasana (standing poses, including toe fan, warrior, tree pose) (9 minutes)<br>6) Supine (twists, bridge) (3 minutes)	Physical function- The Timed Up and Go (TUG)<br>Balance/stability- The Functional Reach (FR) test<br>Flexibility- The Sit and Reach Test (SR)<br>Pain- Brief Pain Inventory (BPI), Patient Neurotoxicity Questionnaire (PNQ) and Functional Assessment of Cancer Therapy—Neurotoxicity (FACT-GOG-NTX)<br>Stress- Perceived Stress Scale (PSS) and Salivary cortisol<br>Sleep- Pittsburgh Sleep Quality Index (PSQI)<br>Spirituality- Functional Assessment of Chronic Illness Therapy—Spiritual Well-being Scale (FACIT-SP)<br>Fear of falling- Falls Efficacy Scale (FES)<br>Vibration sense- measured on the feet through a calibrated bioesthesiometer<br>Patient experiences- observations and focus group	SR- significant improvement in flexibility (mean increase = 8.53; SD = 7.52; *P* = .006)<br>FR- significant improvement in balance (mean increase = 14.32; SD = 8.78; *P* = .001)<br>TUG- improved significantly, indicating improved mobility and balance and reduction in fall risk (mean reduction = 2.11, SD = 1.75, *P* = .004)<br>BPI- significant reduction in pain severity (4.84 to 2.90, *P* = .041) and in pain interference (4.77 to 3.18, *P* = .011)<br>PNQ- significant improvement in sensory symptoms and muscular weakness (6.8 to 5.3, *P* = .003)<br>FACT-GOG-NTX- not significant but demonstrated trends of improvement (95.90 to 104.4, *P* = .445)<br>PSS- stress was reduced (18.8 to 14.8, *P* = .056)<br>PSQI- sleep quality non-significantly improved (10.5 to 8.9, *P* = .23)

			7) iRest® Yoga Nidra (20 minutes)<br>8) Savasana (5 minutes)<br>9) Postpractice body scan (2 minutes)<br>10) Seated:<br>- Mandala mudra with affirmation, sun breath, Anjali mudra, aum, namaste (2 minutes)<br><br>Frequency: twice per week<br><br>Intensity: N/A<br><br>Time, 180mins per week, 8-weeks<br><br>In-person (supervised) and home-based (unsupervised)		FACIT-SP- spirituality improved non-significantly (103 to 110, *P* = .510)<br><br>FES- fear of falling non-significantly reduced (37.7 to 28.8, *P* = .07)<br><br>Salivary cortisol- reduced non-significantly (0.131 to 0.118, *P* = .12)<br><br>Sensation as measured via bioesthesiometer- improvement in all tested locations on the feet, with a single location (tip of right great toe) revealing statistically significant improvement (mean improvement = 8.2, SD = 10.6, *P* = .035)<br><br>Qualitative- themes: (1) vacillation of CIPN pain perception over time; (2) transferability of skills to daily activities learned throughout the yoga intervention; (3) improvement in physical function, leading to return to various work and hobbies; (4) perceived relaxation as an effect of the yoga and meditation; and (5) group engagement providing a social context for not feeling isolated with CIPN.
[23] Ikio et al., 2022	Single-blind, single-centre, randomised controlled trial<br><br>Sample size: n = 39	Males and females:<br>M = 22<br>F = 17<br><br>Age: 57-89 years<br><br>Type of cancer: hematologic, gastrointestinal	Type: multimodal program:<br>Muscle strength exercises: grip and pinching movements using hand and finger exerciser<br>Manual dexterity training: origami and paper tearing<br>Sensory function training: material identification and tactile perception practice	Upper extremity function: Michigan Hand Outcomes Questionnaire (MHQ)- (1) overall hand function, (2) activities of daily living (ADL), (3) work performance, (4) pain, (5) aesthetics, and (6) patient satisfaction with hand function<br><br>Muscle strength: hand dynamometer	In the intention-to-treat analysis:<br>The ADL of MHQ decline was significantly more suppressed in the intervention group (IG) than the control group (CG) at T2 (difference: 7.23; 95% confidence interval [CI]: 0.35–14.10; P = 0.0397). There were no significant differences between the groups in other MHQ subscales. The suppression of ADL of MHQ decline was significantly higher in the IG than

			Frequency: at least 3 times per week<br>Intensity: 40-60% of maximum muscle strength<br>Time: 30mins<br>Unsupervised	Sensory function: Semmes–Weinstein monofilament test<br>Manual dexterity: Purdue Pegboard test<br>Symptom severity: visual analogue scale (VAS)<br>Quality of life: Functional Assessment of Cancer Therapy/Gynecologic Oncology Group-Neurotoxicity (FACT/GOG-Ntx) questionnaire<br>Pain catastrophising: pain catastrophising scale (PCS)	in the CG at T1 (difference: 6.79; 95% CI: 0.30–13.28; P = 0.0405).<br>The pinch strength in the IG was significantly improved than in the CG at T1 (difference: 0.86; 95% CI: 0.37–1.34; P = 0.0007) and at T2 (difference: 0.69; 95% CI: 0.17–1.21; P = 0.0096).<br>The CG performed significantly better than the IG in the Purdue Pegboard test than the IG (difference: − 1.1; 95% CI: − 2.06– − 0.13; P = 0.0266) at T2.<br>The social/family subscale of FACT-COG/Ntx significantly improved in the CG than in the IG (difference: − 2.83; 95% CI: − 5.25– − 0.40; P = 0.023) at T1.<br><br>In the as-treated analysis:<br>The ADL of MHQ decline was significantly more suppressed in the IG than the CG at T2 (difference: 13.09; 95% CI: 5.68–20.49; P = 0.0008).<br>There was a significantly greater improvement in pain of MHQ in IG than the CG at T2 (difference: 13.21; 95% CI: − 22.91 to − 3.51; P = 0.0083).<br>The total MHQ score decline was significantly more suppressed in the IG than in the CG at T2 (difference: 7.6; 95% CI: 0.71–4.49; P = 0.0312).<br>ADL of MHQ decline was significantly more suppressed in the IG than in the CG at T1 (difference: 10.61; 95% CI: 3.29–17.93; P = 0.0051).<br>There was a significantly greater improvement in pain of MHQ in IG than the CG at T1 (difference: − 12.24; 95% CI: − 21.82 to − 2.66; P = 0.013).

					When compared to the CG, the IG showed significant improvements in the total MHQ score at T1 (difference: 8.38; 95% CI: 1.56–15.2; P = 0.0169). The improvement in pinch strength was significantly greater in the IG than in the CG at T2 (difference: 0.76; 95% CI: 0.18–1.34; P = 0.0115).
[24] Kneis et al., 2019	Randomised controlled trial<br><br>Sample size: n = 41	Males and females:<br>M = 11<br>F = 30<br><br>Age: 44-82 years<br><br>Type of cancer: breast, colorectal, gynaecological, upper gastrointestinal, non-small cell lung, non-Hodgkins's lymphoma, multiple myeloma	Type: multimodal program-<br>Endurance exercise: stationary bike<br>Balance training: 3-8 exercises with 3 repetitions each 20 – 30secs, progressively reducing support surface and visual input, adding motor/cognitive tasks, and instability induction<br><br>Frequency: twice per week<br><br>Intensity: Moderate intensity/below the individual anaerobic threshold<br><br>Time: 12 weeks, 60mins<br><br>Note: control group completed endurance intervention but not balance intervention.	Functional performance:<br>Balance- centre of force sway path on force plate during semi-tandem stance with eyes open/closed, and mono-pedal stance.<br>Single leg stance test on stable and unstable surfaces.<br>Muscular power- maximum counter-movement jump<br><br>Quality of life: European Organization for Research and Treatment of Cancer Quality of Life (EORTC QLQ-C30) questionnaire<br><br>Symptom severity: EORTC QLQ-CIPN20 and neurotoxicity subscale (NtxS) of Functional Assessment of Cancer Therapy/Gynaecology Oncology Group (FACT&GOG)<br><br>Vibration sense: Rydel-Seiffer tuning fork<br><br>Cardiorespiratory fitness: peak oxygen consumption, maximum power output and performance at the individual anaerobic threshold during maximum cardiopulmonary exercise test	In the per-protocol analysis:<br>IG's semi-tandem stance with eyes open sway path decreased significantly (− 76 mm, 95%CI -141 – -17; *p* = .018), while the CG's was unchanged. There was a significant difference in groups' delta (*p* = .049).<br>There were no inter- or intra-group changes in semi-tandem stance with eyes closed.<br>Both groups improved descriptively in the mono-pedal stance with eyes open. However, this was not statistically significant, but there were moderate effect sizes ($r_\varphi = 0.41$; $r_\varphi = 0.51$, respectively).<br>Only the IG improved in single leg stance test (Stable surface: 1 s, 95%CI 0–7; *p* = .051; Unstable surface: 11 s, 95% CI 8–17; *p* = .001). There was a significant difference in groups' delta for unstable surface (*p* = .000).<br>CG had significant improvements to maximum jump height (2 cm, 95%CI 0.5–3.5; *p* = .039), while the IG did not change. Maximum power did not change.<br>There were no inter- or intra-group differences in vibration sense at the first metacarpophalangeal joint. At the knuckle, the CG increased significantly

					(0.8, 95% CI 0.3–1.3; *p* = .011), resulting in a significant group difference at T1 (*p* = .049). There was a significant improvement in vibration sense at the patella for the CG (1.0, 95% CI 0.4–1.6; *p* = .002), while the IG decreased significantly (− 0.8, 95% CI -0.2 – 0.0; *p* = .041), resulting in a significant difference at T1 (*p* = .005) and in groups' delta (*p* = .000).<br>The IG reported significantly alleviated symptoms in the NtxS (3, 95% CI 1–6; *p* = .015). CIPN20 showed a significant reduction in symptoms in the IG, except for the upper extremity sub-score (sum score: -10, 95% CI -17 – -4; *p* = .007; sensory score: -7, 95% CI -15 – 0; *p* = .028; motor score: -8, 95% CI -18 – 0; *p* = .006; autonomic score: -8, 95% CI -17 – 0; *p* = .006; lower extremity score: -13, 95% CI -19 – -4; *p* = .007). The CG's sum, sensory and lower extremity scores also decreased significantly (− 6, 95% CI -11 – -1; *p* = .027; − 7, 95% CI -15 – 0; *p* = .018; − 8, 95% CI -15 – -2; *p* = .014; respectively). Both groups' global QoL improved, but this change was not significant.<br>The CG significantly improved individual anaerobic threshold post-intervention (0.1 W/kg, 95%CI 0.0–0.1; *p* = .020). There was no change for IG (*p* = .122). Maximum power output improved for both groups (IG: 0.1 W/kg, 95%CI 0.0–0.2; *p* = .025; CG: 0.1 W/kg, 95%CI 0.0–0.2; *p* = .004). There were no

					differences in peak oxygen consumption.
[31] Schwenk et al., 2016	Single blinded, randomised, controlled trial.<br><br>Sample size: n = 22	Males and females:<br>M = 9<br>F = 13<br><br>Age: 55-86 years<br><br>Type of cancer: lung, multiple myeloma, breast, colorectal, melanoma, bladder, prostate, pancreas, ovarian, chronic lymphoid leukemia	Type: Interactive sensor-based balance training- repetitive, error-dependent forward/backward/sideward/diagonal leaning tasks and cognitively challenging dynamic weight shifting tasks using wearable sensors<br><br>Frequency: twice per week<br><br>Intensity: N/A<br><br>Time: 4 weeks, 45mins<br><br>Supervised, face-to-face	Balance- centre of mass sway measured using wearable sensors while participants are in double leg or semi-tandem stance with eyes open or closed.<br><br>Gait performance- speed and variability measured using wearable sensors while participant performs 10m walk<br><br>Fear of falling- Falls Efficacy Scale (FES-I)	Medial-lateral centre of mass sway, hip sway and ankle sway were reduced in the IG compared to CG during balance assessments with double leg stance and eyes open (p= .010– .022).<br><br>Significant reductions in postural sway were also found in semi-tandem stance (p= .008– .035), except for ankle sway (p= .294). Greatest effects were found for hip sway during semi-tandem stance (η_p^2= .388; −74.8%).<br><br>Medial-lateral centre of mass sway, hip sway and ankle sway during double leg stance with eyes closed were improved in the IG by 34.9%, 39.1%, and 24.7% respectively. Gait speed was improved in the IG by 8.2%. However, these were not significant. The effect sizes for these outcomes suggest presence of a moderate effect (η_p^2= .037– .078).
[32] Streckmann et al., 2019	Prospective, single-center, four-armed randomised controlled trial<br><br>Sample size: n = 40	Males and females:<br>M = 13<br>F = 27<br><br>Age: 47-70 years<br><br>Type of cancer: breast, ovarian, adenocarcinoma, colon, pancreatic, T cell NHL, M. Hodgkin, plasmacytoma, multiple myeloma rectal, lung	Type: sensorimotor training- progressively more difficult balance exercises on progressively unstable surfaces<br><br>Frequency: twice per week<br><br>Intensity: N/A<br><br>Time: 6-week program, each exercise performed 3 times for 20sec with a rest of 40sec between each set and 1 min between each exercise<br><br>Supervised	CIPN clinical test battery:<br>Achilles and patella tendon reflexes: using reflex hammer<br>Peripheral deep sensitivity: Rydel-Seiffer tuning fork<br>Light-touch perception: researcher stroking feet and legs<br>Sense of position: researcher changing position of toes<br>Lower leg strength: manual muscle test<br>Patient- reported CIPN symptoms: FACT GOG-Ntx questionnaire	Sensorimotor training group (STG) showed significantly higher improvements than control group (CG) in:<br><br>Patella tendon reflex- (SMT vs CG *P* = .020, *d* = 1.514)<br><br>Achilles tendon reflex- (SMT vs CG *P* = .042, *d* = 0.978/SMT vs WBV *P* = .036, *d* = 0.978)<br><br>FACT GOG-Ntx questionnaire-(SMT vs CG *P* = .096, *d* = 1.079)

				Nerve conduction velocity: sensory nerve action potentials recorded with electrodes<br>Balance control: force plate<br>Health-related quality of life: EORTC-QLQ-C30 questionnaire<br>Neuropathic pain: Pain-DETECT questionnaire	
[25] Vollmers et al., 2018	Randomised prospective and controlled intervention trial<br>Sample size: n = 36	Females<br>Age: 20.08-68.67 years<br>Type of cancer: primary breast	Type: multimodal program-<br>Static and dynamic sensorimotor training: single leg stance and smovey exercises on posturomed devices<br>Upper and lower extremity strength training: 6 exercises performed at 2 sets of 20 repetitions<br>Endurance training: warm-up<br>Frequency: twice per week<br>Intensity: RPE of 13–15 (moderate to rather difficult) on the Borg Scale<br>Time: 18 weeks	Quality of life: EORTC QLQ-C30 questionnaire and EORTC BR23 questionnaire<br>Symptom severity/pain: EORTC CIPN20 questionnaire<br>Postural stability: Fullerton Advanced Balance Scale<br>Upper extremity strength: hand dynamometer<br>Lower extremity strength: Chair Rising Test.<br>Fatigue: MFI-20 questionnaire	No significant improvements in psychological parameters, adverse events and Chair Rising Test in intervention group (IG) compared to control group (CG).<br>Significantly smaller sway area in mono-pedal stance in IG compared to CG at T1 [left leg 16.17 ± 3.67 vs. 21.55 ± 5.33 ($p < 0.001$) and right leg 15.14 ± 2.30 vs. 20.85 ± 5.05 ($p < 0.001$)] and T2 [left leg 15.22 ± 3.86 vs. 20.75 ± 6.89 ($p = 0.003$) and right leg 14.81 ± 3.53 vs. 20.95 ± 6.33 ($p < 0.01$)]. Significant improvement over time in bipedal stance [T1 vs. T0, − 0.49 (IG) vs. + 1.14 (CG) $p = 0.039$].<br>Significant differences in Fullerton Advanced Balance Scale [37.71 ± 2.73 vs. 34.47 ± 3.98 ($p = 0.004$)]. CG decreased between T0 and T1 while IG improved slightly [+ 1.35 (IG) vs. − 2.84 (CG) ($p < 0.001$)].<br>Significant loss of strength in CG compared to slight improvement in IG (− 1.60 vs. 0.60, $p = 0.029$).

[26] Zimmer et al., 2018	Randomised controlled trial with two-armed, monocentric design<br><br>Sample size: n = 30	Males and females:<br>M = 21<br>F = 9<br><br>Age: 50-81 years<br><br>Type of cancer: metastasised colorectal cancer	Type: multimodal program:<br>Balance training: balance pads, balancing on lines<br>Coordination practices: cherry pit pillows, Brasils<br>Endurance training: cross trainer, bicycle ergometer or walking<br>Resistance training: circuit training- bench press, lat pulldown, leg press, seated row, abdominal exercise- 2 sets of 8-12 repetitions<br>Cool down: relaxing, stretching, breathing and mobilisation exercises<br><br>Frequency: twice per week<br><br>Intensity:<br>Endurance training: RPE of 12-13 on Borg scale/60-70% of maximum heart rate<br>Resistance training: Borg CR10 scale level 6/weight: 60-80% of H1RM<br><br>Time: 8 weeks, 60mins<br><br>Supervised, face-to-face	Symptom severity/quality of life- Trial Outcome Index (TOI) of the Functional Assessment of Cancer Therapy/Gynaecologic Oncology Group Neurotoxicity (FACT/GOG-NTX) questionnaire<br><br>Balance- Gleichgewichtstest (GGT-Reha) including static and dynamic stances, and unstable surfaces<br><br>Dynamic muscular strength- hypothetic one-repetition maximum (h1RM) bench press, leg press and lat pulldown<br><br>Endurance capacity- six-minute walk test (6MWT)	Symptom severity/quality of life: there were significant differences in TOI between the IG and CG from baseline to t_1 and from baseline to t_2 whereas no significant difference was observed from t_1 to t_2. TOI remained stable over time in the IG, whereas it decreased from baseline to t_1 by an average of −7.14 (median: −4) points (p = .077) and significantly decreased from baseline to t_2 by −8.07 (−8) points (p = .037) in the CG. Only the neurotoxicity subscale showed significant differences over time between IG and CG. The IG had significant improvements by 2.12 (2) points from t_0 to t_1 (p = .023) whereas neuropathic symptoms significantly worsened in the CG (−5.11 (−3) points; p = .045). CG had a reduction of 4.65 (3) points from t_0 to t_2 (p = .054).<br><br>Balance: there were no significant differences in simple static and dynamic balance between groups over time. There were significant differences between IG and CG in advanced static balance from baseline to t_1 and from baseline to t_2 whereas no significant difference was observed from t_1 to t_2, whereby IG improved from baseline to t_1 by 1.88 (2) points (p = .048)<br><br>Strength: There were significant differences between groups in all muscle groups from baseline to t_1 and from baseline to t_2 whereas no significant differences were observed

					from t_1 to t_2. IG increased in strength from baseline to t_1 in the three muscle groups (bench press: 38.06 (37.21) kg, $p = .006$; leg press: 38.73 (19.84) kg, $p = .002$; lat pulldown: 8.76 (6.92) kg, $p < .001$). From t_1 to t_2 leg press improved by – 1.22 (+5.92) kg ($p = .005$) whereas bench press remained stable ($p = .069$) and lat pulldown decreased (−2 (−3.89) kg, $p = .008$). CG only improved in lat pulldown from t_0 to t_1 by 2.44 (2.11) kg ($p = .001$).<br><br>Endurance: There were no significant differences in endurance capacity between groups. IG improved walking distance by 24.44 (14) m while CG improved by 18.5 (21) m.<br><br>There was a significant association between changes in TOI as well as NTX score and advanced static balance (TOI - balance, $p = .022$; $r = .417$, NTX - balance, $p = .006$; $r = .488$).
